# Recurrent Symptomatic Hyponatremia Revealing a Late Diagnosis of Sheehan Syndrome and Effectively Treated With Levothyroxine: A Case Report and Brief Review of Literature

**DOI:** 10.1002/ccr3.71285

**Published:** 2025-10-15

**Authors:** Hayatu Awel Abdela, Germen Temam Abdulmalik, Numeri Husein Kimo, Kumel Nur Muktar, Rebi Ali Oumer, Tamirat Godebo Woyimo

**Affiliations:** ^1^ Department of Internal Medicine, School of Medicine, College of Medicine and Health Sciences Wolkite University Wolkite Ethiopia; ^2^ Department of Clinical Radiology, School of Medicine, College of Medicine and Health Sciences Wolkite University Wolkite Ethiopia; ^3^ Department of Pediatrics and Child Health, School of Medicine, College of Medicine and Health Sciences Wolkite University Wolkite Ethiopia; ^4^ Department of Obstetrics and Gynecology, School of Medicine, College of Medicine and Health Sciences Wolkite University Wolkite Ethiopia; ^5^ Department of Internal Medicine, School of Medicine, College of Medicine and Health Sciences Jimma University Jimma Ethiopia

**Keywords:** hormone replacement therapy, hyponatremia, hypopituitarism, levothyroxine therapy, secondary hypothyroidism, Sheehan syndrome

## Abstract

In women with a history of postpartum hemorrhage presenting with chronic symptoms such as hyponatremia, amenorrhea, weight loss, or cold intolerance, suspect Sheehan's syndrome. Early diagnosis and prompt hormonal replacement therapy are essential for reversing complications and improving quality of life. Prioritize evaluation of pituitary function in these patients to ensure timely intervention.

## 1 Introduction

Sheehan's syndrome (SS) is a rare yet serious cause of hypopituitarism due to ischemic necrosis of the pituitary gland after postpartum hemorrhage [1]. It remains a leading etiology in low‐resource settings—accounting for 6%–8% of hypopituitarism cases globally and reaching a prevalence of 3.1% in some regions [1, 2]. While the acute form is well characterized, chronic, late‐onset SS—especially presenting with recurrent hyponatremia—is often overlooked. Recent cases have broadened the clinical spectrum: a 2024 report described hyponatremic rhabdomyolysis, and a 2025 case featured diagnosis 40 years after hemorrhage—among the longest delays recorded [3, 4].

Multicenter data indicate diagnostic delays averaging ~9 ± 9.7 years in high‐income countries and exceeding 20 years in resource‐limited regions, with delays of over 30 years reported [5, 6, 7]. SS may also manifest with anemia, hypoglycemia, and neuropsychiatric symptoms—underscoring its diverse clinical face [8]. These observations highlight an expanding and atypical presentation of SS and reinforce the novelty of our case and the importance of awareness of SS's diverse presentations.

Despite this, chronic SS with hyponatremia remains under‐recognized, reflecting a persistent knowledge gap. Clinicians may not suspect SS when confronting nonspecific symptoms like fatigue and hyponatremia in women with remote obstetric hemorrhage. To bridge this gap, we present a case report describing the diagnostic work‐up—including pituitary axis assays and differentials—and emphasize how timely hormone replacement led to rapid clinical improvement. By contextualizing this case within contemporary literature, we aim to raise awareness and encourage earlier endocrine testing in at‐risk women.

## 2 Case Presentation

A 42‐year‐old female presented to the emergency department with a 1‐week history of nausea, vomiting, global headache, and confusion. She also reported significant loss of appetite, asthenia, and an inability to ambulate or perform routine activities, including self‐care, without assistance. This was her third hospitalization for similar symptoms, having been admitted twice in the preceding 2 months with severe chronic symptomatic hyponatremia (serum sodium levels of 108 and 110 mEq/L) of unknown etiology, treated with hypertonic saline.

Upon further questioning, the patient disclosed a 2‐year history of decreased appetite, significant weight loss, easy fatigability, and constipation, with bowel movements occurring only once or twice every 2 weeks. She also reported sensitivity to cold and anhedonia. The patient is a Para‐three mother with two living children aged 16 and 18, alongside one early neonatal death. She has experienced amenorrhea for the past 15 years following a complicated home delivery of triplets that resulted in massive postpartum hemorrhage requiring multiple units of blood transfusion and leading to immediate neonatal death. Five years prior, she sought infertility treatment at a local primary care facility where she was prescribed combined oral contraceptives (COC) for 3 months, which temporarily restored her menstrual cycle (Table 1).

**TABLE 1:** Timeline of key clinical events: summarizes the patient's clinical course with exact years estimated from the history.

Year	Clinical event or intervention	Notes/Clinical impact
2009	Triplets pregnancy complicated with postpartum hemorrhage	Massive postpartum hemorrhage (PPH) requiring multiple transfusions led to the immediate death of all three neonates
2009	Onset of secondary amenorrhea	After the episode of postpartum hemorrhage, the patient did not have any menses, which persisted for 15 years
2019	Infertility consultation and treatment	Patient sought infertility care; given combined OCPs which temporarily restored menstruation
2022	Gradual onset of hypothyroid symptoms	Two‐year history of fatigue, significant weight loss, cold intolerance and constipation developed
2024	Multiple emergency department visits (due to hyponatremia)	Three hospitalizations over 2 months for severe symptomatic hyponatremia (serum Na ≈ 108–110 mEq/L), treated with hypertonic saline
2024	Diagnosis of Sheehan's syndrome	Based on remote history of PPH, prolonged amenorrhea, pituitary hormone deficiencies and MRI showing empty sella
2024	Initiation of levothyroxine therapy	Started on low‐dose oral levothyroxine (1.6 μg/kg) after hyponatremia correction
2024	Clinical improvement and resolution	Six months posttreatment: normalized sodium, full resolution of hypothyroid symptoms (weight gain, normal bowel habits, improved energy and cold intolerance)

*Note:* Key events (years) and interventions are aligned with the original case details.

On physical examination, the patient appeared acutely ill against a background of chronic illness. She was emaciated, weighing 37 kg with a body mass index (BMI) of 13.59 kg/m^2^. Vital signs were as follows: blood pressure ranged from 90–135/60–81 mmHg, pulse rate between 56 and 65 bpm, respiratory rate from 14 to 20 bpm, temperature between 35°C and 36°C, and oxygen saturation at 94%–96% on room air. There was no evidence of thyroid enlargement; her skin was dry and cold, and there was no edema present. The patient exhibited confusion and disorientation to time and place, with a Glasgow Coma Scale (GCS) score of E4V4M6, but no focal neurological deficits were noted.

## 3 Differential Diagnosis

The primary differential diagnoses considered included postpartum pituitary apoplexy, spontaneous infarction of a silent adenoma, lymphocytic hypophysitis, and primary empty sella syndrome (ESS). Postpartum pituitary apoplexy was less likely due to the delayed onset of symptoms and absence of acute neurological deficits [6]. Spontaneous infarction of a silent adenoma was improbable given the lack of a visible pituitary mass on MRI and the clear history of postpartum hemorrhage. Lymphocytic hypophysitis, typically presenting with pituitary enlargement and headaches in the peripartum period, was ruled out due to the absence of these features [9]. Primary ESS, characterized by the herniation of subarachnoid space into the sella turcica, could explain some MRI findings but was likely secondary to Sheehan's syndrome given the significant postpartum hemorrhage and progressive pituitary dysfunction observed. Primary adrenal insufficiency and SIADH were also considered. Adrenal failure was excluded by a normal morning cortisol and electrolyte profile. SIADH was deemed unlikely given the clinical context. Ultimately, our patient met the established criteria for Sheehan's syndrome (history of postpartum hemorrhage, ≥ 2 pituitary hormone deficits, and empty sella on MRI).

## 4 Investigations

A comprehensive workup was conducted, including complete blood count (CBC), random blood sugar (RBS), urinalysis (U/A), renal function tests (RFT), stool microscopy, liver function tests (LFT), liver enzymes, HIV testing, HBsAg, and HCV‐RNA, all of which returned within normal range. Serum sodium was significantly low (108 mEq/L), with lower serum osmolality (235 mOsm/kg). However, due to the absence of investigation in our hospital, urine sodium value and urine osmolality were not conducted. A 12‐lead electrocardiogram (ECG) indicated sinus bradycardia (55–65 bpm), while a two‐dimensional transthoracic echocardiography (TTE) revealed minimal pericardial effusion, otherwise normal findings. Abdominal ultrasound and upper gastrointestinal endoscopy were unremarkable. A serum pituitary hormonal evaluation suggested anterior pituitary hormone deficiency (central hypothyroidism, lactotroph deficiency, and secondary gonadal dysfunction) (Table 2).

**TABLE 2:** Laboratory results during hospital stay and follow‐up.

Test (reference range)	1st visit	2nd visit	3rd visit	Follow‐up (6th month)
Serum electrolyte				
Na (135–145 mEq/L)	108	110	103	137
K (3.5–5.1 mEq/L)	3.6	4.2	3.78	4.0
Cl (98–107 mEq/L)	89	93	71.6	103
Hormone tests				
TSH (0.3–4.2 pmol/L)	3.74		2.86	1.94
fT3 (2.8–7.1 pmol/L)	3.41			
fT4 (12–22 pmol/L)	2.86			17.18
Total T4 (66–181 nmol/L)			22.66	
8:am cortisol (201–536 nmol/L)			> 1000, 654	
Prolactin (5–35 ng/mL)			3.26	
FSH for menopause women (22.7–130 IU/mL)			17.04	
LH for menopause women (11–40 IU/mL)			11	
Other tests				
Complete blood count	Normal	Normal	Normal	
Random blood sugar (mg/dL)	111	115		
Urine specific gravity	1.005	1.015	1.010	
Creatin (0.5–0.9 mg/dL)	0.66		0.71	
Urea (16.6–48.5 mg/dL)	23		21	
AST (0–40 IU/L)	143			22.6
ALT (0–41 IU/L)	37			36.47
ALP (40–130 IU/L)	195			124.66
LDH (140–280 IU/L)	259			202
HIV	NR			
HBsAg	NR			
HCV‐RNA	NR			
VDRL	NR			
Imaging				
Brain MRI	Empty sella			
Abdominal US and upper GI endoscopy	Unremarkable			
Echocardiography	Minimal pericardial effusion, otherwise unremarkable			

Abbreviations: ALP, alkaline phosphatase; AST/ALT, aspartate transaminase/alanine transaminase; CBC, complete blood count; FSH, follicle‐stimulating hormone; fT3, free triiodothyronine; fT4, free thyroxine; GH, growth hormone; HIV, human immunodeficiency virus; LH, luteinizing hormone; NR, nonreactive; RBS, random blood sugar; s.g, specific gravity; TSH, thyroid stimulating hormone; VDRL, Venereal Disease Research Laboratory.

### 4.1 Diagnostic Evaluation

**FIGURE 1:**
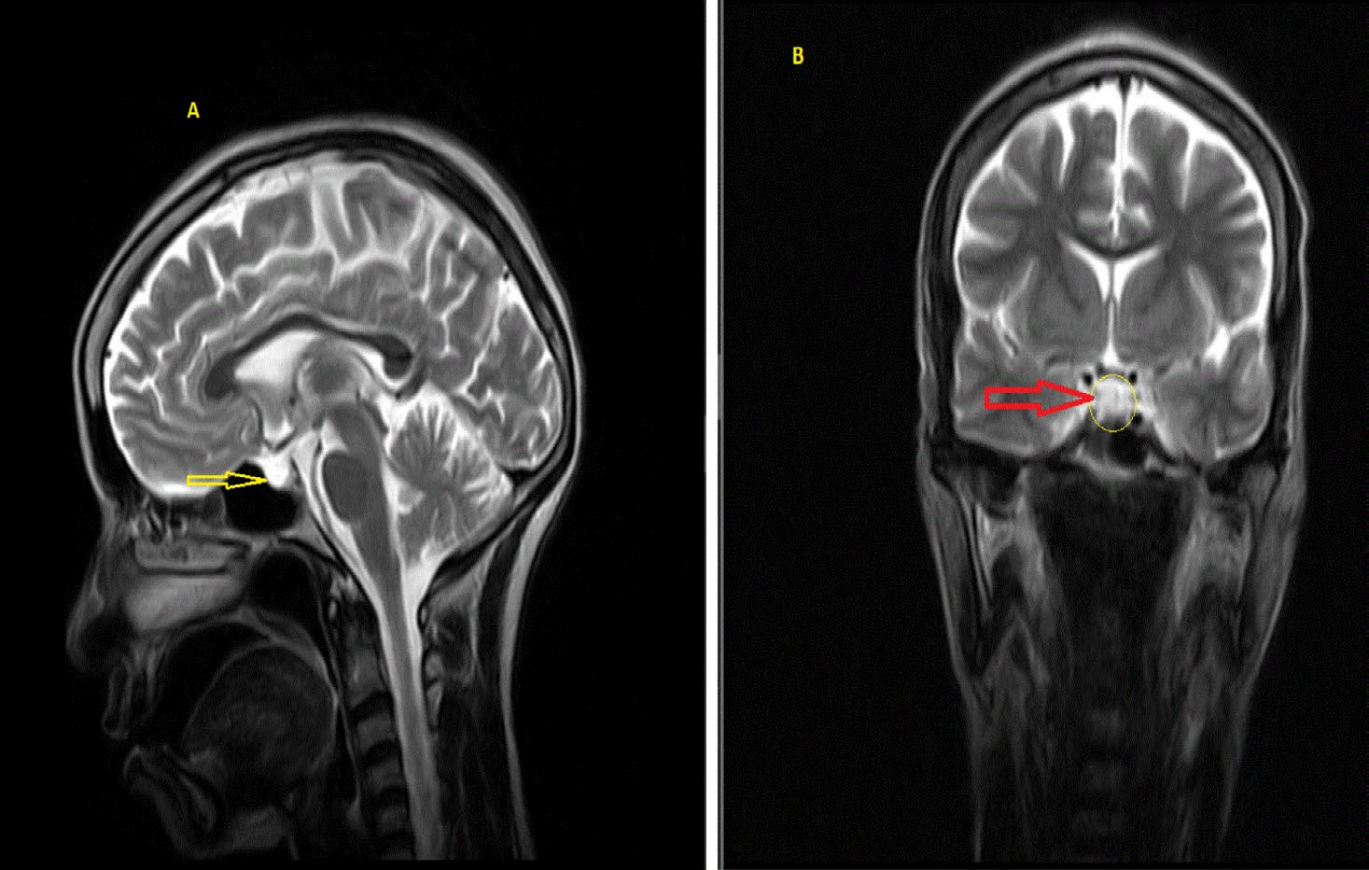
T2‐weighted MRI of the pituitary fossa. (A) Sagittal plane showing the sella turcica region. Note the pituitary tissue is compressed to the sellar floor, with increased cerebrospinal fluid (CSF) signal filling the pituitary fossa (yellow arrow), consistent with a Grade 4 empty‐sella appearance. (B) Coronal plane demonstrating near‐total absence of pituitary tissue, with CSF occupying most of the sella (red arrow and yellow circle). No cystic changes or mass lesions are present.

**FIGURE 2:**
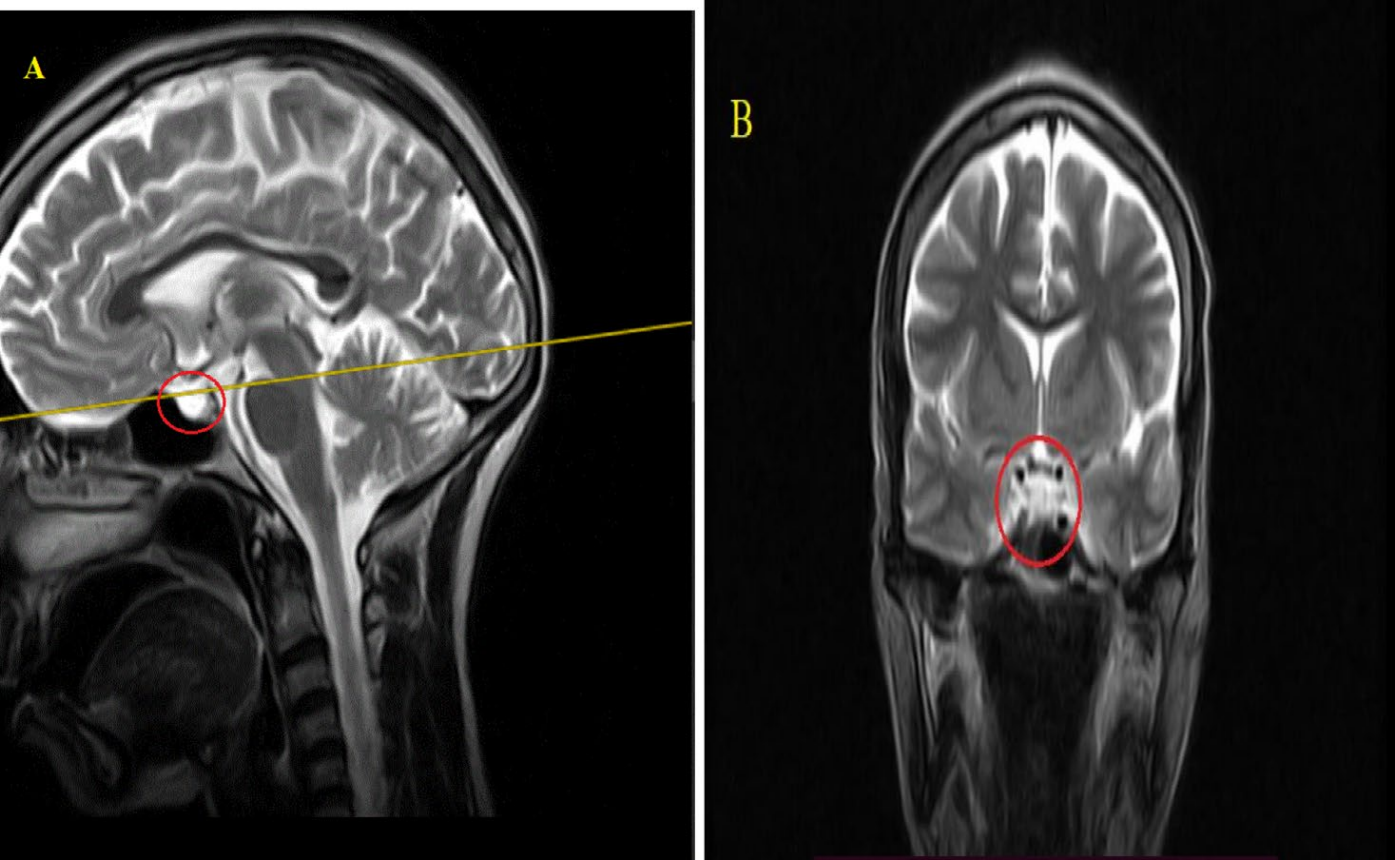
Additional T2‐weighted MRI views of the sellar region. (A) Sagittal view repeats anatomical context and confirms CSF‐filled sella space (red circle). (B) Coronal view again reveals minimal pituitary tissue with CSF predominance, consistent with significant pituitary atrophy posthemorrhage (red circle). CSF, cerebrospinal fluid.

We used Kelestimur's revised criteria—history of postpartum hemorrhage, multiple pituitary hormone deficiencies, and radiologic empty sella—to confirm chronic SS [10]. Specifically, our patient's endocrine testing showed: central hypothyroidism (low free T4 with inappropriately normal TSH), hypogonadotropic hypogonadism (low FSH/LH consistent with 15 years of amenorrhea), and preserved adrenal axis (normal 8 AM cortisol; > 3 μg/dL), ruling out secondary adrenal insufficiency [2, 11, 12]. Growth hormone assessment was unavailable. MRI demonstrated an empty sella, supporting significant pituitary atrophy (Figures 1 and 2, Video 1). These findings—when combined with obstetric history—satisfy established diagnostic parameters for chronic SS. Additionally, in SS, GH and prolactin are most commonly affected, with TSH and ACTH deficiencies seen in up to 100% and 56%–100% of cases respectively; preservation of cortisol in our patient is therefore noteworthy [10].

**VIDEO 1:** Sagittal sweep through the sella turcica: a high‐resolution dynamic sagittal sequence that reproduces and clarifies the static images in Figures 1 and 2. It demonstrates compression of the pituitary gland against the sellar floor with homogeneous cerebrospinal fluid signal filling and distending the pituitary fossa (annotated arrow/circle), consistent with a Grade 4 empty‐sella. The continuous cine/scroll allows real‐time assessment of gland contour, CSF occupancy and adjacent anatomy to aid clinician interpretation. Video content can be viewed at https://onlinelibrary.wiley.com/doi/10.1002/ccr3.71285.

## 5 Treatment, Outcome and Follow‐Up

Based on the clinical history, hormonal profile, and imaging findings, a diagnosis of Sheehan's syndrome was made. Following correction of hyponatremia with hypertonic saline infusion, the patient was started on a lower dose of oral levothyroxine (1.6 μg/kg) and referred to the outpatient department for regular follow‐ups.

After 6 months of outpatient follow‐up with monitoring of free T4 levels and levothyroxine dose adjustments, the patient demonstrated significant clinical improvement with no episode of hyponatremia. Symptoms of hypothyroidism resolved completely; she experienced improved cold intolerance, and her skin became well‐moisturized. Bowel movements normalized from once every 2 weeks to every other day. Additionally, her interest in family and social activities improved markedly, allowing her to resume routine activities, including her job as a merchant. She was also advised of the need for lifelong endocrine follow‐up.

## 6 Conclusion

This case underscores the importance of considering Sheehan's syndrome in women with a history of postpartum hemorrhage and chronic symptoms of pituitary dysfunction. Early recognition and appropriate hormonal replacement therapy can significantly improve patient outcomes and quality of life. This case highlights the importance of obtaining an obstetric history in women with unexplained hyponatremia or chronic pituitary symptoms, even decades after delivery. We show that even very delayed SS can be effectively treated: after 6 months of appropriate therapy, our patient's metabolic and neurological symptoms had completely reversed. Delays in SS diagnosis lead to significant morbidity and reduced quality of life, and untreated late‐onset SS may even be fatal. This report underscores that early recognition and prompt multi‐hormonal replacement are critical for improving outcomes in patients with SS.

## 7 Discussion

Sheehan's syndrome (SS) is a rare yet significant condition resulting from severe blood loss and hypovolemic shock during or after childbirth, leading to ischemic necrosis of the pituitary gland. During pregnancy, the pituitary gland enlarges due to hyperplasia of lactotroph cells in response to estrogen stimulation [9]. This increased size, coupled with the gland's high vascularity, makes it particularly susceptible to ischemia during episodes of sudden blood volume depletion. The pathogenesis of SS is multifactorial, involving increased pituitary size, a small sella turcica, autoimmune responses against pituitary antigens, hypercoagulation leading to thrombosis of pituitary arteries, and vasospasm of these vessels [13, 14]. Notably, Karaca and Kelestimur recently reviewed SS and emphasized that, despite its rarity, SS remains common in underdeveloped countries and often goes unrecognized, leading to long diagnostic delays [10]. Osorio‐Toro et al. reported a case diagnosed 38 years post‐delivery, underscoring how indolent the disease can be [2]. Laway and Baba [15] have also highlighted long‐term sequelae of untreated SS, showing increased cardiometabolic risk (e.g., dyslipidemia, insulin resistance) that may be improved with growth hormone and another hormone replacement [15]. These recent reports reinforce the novelty of our case and the importance of awareness of SS's diverse presentations. In this case, the patient's risk factors included a triplet pregnancy, home delivery, and subsequent massive postpartum hemorrhage, all of which likely contributed to pituitary ischemia.

The clinical presentation of SS can vary widely, often mimicking other conditions and leading to misdiagnosis and delayed treatment. Symptoms may manifest immediately postpartum as pituitary apoplexy [6, 16] or lactation failure, or they may be delayed for years or even decades, depending on the extent of anterior pituitary necrosis. Many patients present with chronic and nonspecific symptoms, resulting in diagnostic delays ranging from 1 to 30 years [17, 18, 19]. Recent reports underscore similar diagnostic delays and atypical presentations. Vasconcelos et al. described a 66‐year‐old with SS diagnosed over 30 years after her last delivery [20]. Gao et al. reported severe hyponatremia precipitated by a cardiac intervention—the first such case described in SHS [21]. These examples further highlight the heterogeneous presentations and importance of awareness. In this case, SS was diagnosed 15 years after a complicated childbirth, during which the neonates did not survive, and breastfeeding was not initiated. The first clinical symptom noted was secondary amenorrhea and infertility, which arose 5 years after the postpartum hemorrhage but was initially overlooked. Additionally, during her initial emergency visit, her thyroid‐stimulating hormone (TSH) levels were tested and deemed “normal,” leading to an oversight regarding the possibility of central hypothyroidism. This highlights a common diagnostic challenge in SS—many patients remain asymptomatic during the early stages of the disease.

Patients with SS may experience a range of symptoms due to deficiencies in various anterior pituitary hormones, resulting in conditions such as adrenal insufficiency, hypothyroidism, and gonadal dysfunction. Sanyal et al. observed that only one‐third of patients presented with the classic feature of amenorrhea, while 22% sought emergency care for hyponatremia, 16.7% reported significant weight loss, and 11.1% had elevated TSH levels [18].

On her third emergency visit, a thorough review of her symptoms and obstetric history raised the possibility of SS, prompting a comprehensive evaluation of anterior pituitary hormones. It is important to note that there is no fixed pattern of hormonal deficiency in SS [19, 22]. Typically, deficiencies in lactotrophs and somatotrophs are most prevalent due to their anatomical proximity within the pituitary gland [9].

Sheehan's syndrome diagnosis integrates three essential criteria: (1) documented postpartum hemorrhage, (2) failure to lactate, and (3) persistent amenorrhea/oligomenorrhea. Hormonal confirmation requires ≥ 2 anterior pituitary deficiencies (GH/prolactin most common), supported by MRI findings (empty sella in 70%–80% of chronic cases). Clinical symptoms (fatigue, hypotension) and therapeutic response to hormone replacement further validate the diagnosis, while excluding alternative causes (tumors, autoimmunity). Notably, resource‐limited settings may rely on clinical history and basal hormone testing when MRI is unavailable. Diagnostic delays average 9 years due to nonspecific presentations, necessitating heightened suspicion in women with prior obstetric hemorrhage [10, 23, 24, 25].

In this patient, hormonal testing revealed partial hypopituitarism, characterized by secondary hypothyroidism (evidenced by normal TSH levels alongside markedly low free T4 and total T4 levels), lactotroph deficiency, and hypogonadotropic hypogonadism (indicated by low follicle‐stimulating hormone [FSH], low luteinizing hormone [LH], secondary amenorrhea, and infertility). Notably, adrenal function appeared normal, as demonstrated by consistently normal 8:00 A.M. morning cortisol levels on two separate occasions.

A review of 28 patients with SS found that complete panhypopituitarism—including secondary hypothyroidism, secondary adrenal failure, hypogonadotropic hypogonadism, and growth hormone deficiency—was present in all patients at diagnosis [19]. However, other studies have reported varying degrees of corticotroph function preservation, with 16.7% and 40% [22] of patients exhibiting some level of intact corticotroph activity. It is crucial to recognize that SS may present in emergency settings with severe complications such as coma due to hypothyroidism, hypoglycemia, or shock resulting from adrenal insufficiency [23].

Brain MRI findings in this patient revealed an invisible pituitary gland and intrasellar extension of the subarachnoid space into the sella turcica, with no cystic or other anatomical lesions, consistent with an empty sella (Figures 1 and 2). Radiological evaluations using MRI can show varying changes in the pituitary gland as the disease progresses. Initially, the gland may appear normal or even enlarged due to pregnancy‐related changes but can later shrink and be replaced by cystic structures [26, 27]. Importantly, no direct correlation has been established between MRI findings and the degree of hypopituitarism [27]. In SS, brain MRI often reveals an empty sella (66.7%), partial empty sella (33.3%), or normal sella (71%) [18, 19].

Hyponatremia is the most prevalent electrolyte disorder in SS, affecting approximately one‐third of patients [19, 28]. The chronic development of hyponatremia in SS is thought to result from a combination of factors, including cortisol deficiency, hypothyroidism, volume depletion, and the syndrome of inappropriate antidiuretic hormone secretion (SIADH). The primary mechanism for hyponatremia in chronic hypothyroidism is the decreased capacity for free water excretion due to elevated antidiuretic hormone levels, largely attributed to hypothyroidism‐induced decreases in cardiac output [29].

In this case, the patient experienced recurrent symptomatic hyponatremia, with three episodes occurring over 2 months. Her hyponatremia was effectively managed with levothyroxine alone, suggesting that secondary hypothyroidism was the underlying cause. Patients with SS are advised to have a lifelong follow‐up. Laboratory monitoring should include free T4 (to adjust levothyroxine) and periodic morning cortisol or ACTH‐stimulation testing (to reassess adrenal function). In premenopausal women, sex steroids should be assessed (and replaced) to protect bone. Bone mineral density scans and metabolic screening (fasting lipids/glucose) are recommended given the known SS‐associated osteoporosis and cardiometabolic risks. Patients should be educated about stress‐dose steroids if indicated. Overall, SS patients need regular follow‐up with an endocrinologist, as lifelong hormone replacement and dose adjustments are required [10, 15].

In cases with adrenal insufficiency, glucocorticoids must be given before thyroid hormone to prevent adrenal crisis [10]. Estrogen–progestin therapy would be indicated in premenopausal women to prevent osteoporosis [10]. Growth hormone replacement, though often unavailable locally, should be considered once other axes are replete, since GH deficiency in SS is typically profound and GH therapy has been shown to improve dyslipidemia, bone density, and quality of life [10, 15]. We started levothyroxine at a low dose and titrated by free T4, as recommended especially in older/cardiac patients; GH therapy (if used) would further raise thyroid requirements [10]. Our patient's symptoms (fatigue, constipation, cold intolerance) resolved fully on levothyroxine alone, mirroring the clinical improvement reported in other treated SS cases. Notably, arginine‐vasopressin deficiency is a rare manifestation of SS, and a water deprivation test was not performed. Additionally, both urinary specific gravity and 24‐h urinary volume were within normal ranges, making SIADH unlikely. In conclusion, delays in SS diagnosis lead to significant morbidity. We emphasize that increasing clinician awareness and education about Sheehan's syndrome is essential to prevent missed diagnoses and avoid such delays.

## 8 Strengths and Limitations

The case report offers a thorough evaluation of the patient, showcasing significant clinical improvement following targeted hormonal therapy. However, the study's limitations include the absence of urine sodium, urine osmolality, and growth hormone level measurements due to resource constraints.

## Author Contributions

**Hayatu Awel Abdela:** conceptualization, data curation, formal analysis, investigation, methodology, resources, supervision, visualization, writing – original draft, writing – review and editing. **Germen Temam Abdulmalik:** conceptualization, data curation, investigation, software, visualization, writing – review and editing. **Numeri Husein Kimo:** conceptualization, data curation, resources, software, writing – review and editing. **Kumel Nur Muktar:** conceptualization, data curation, investigation, resources, writing – original draft. **Rebi Ali Oumer:** conceptualization, data curation, formal analysis, validation, writing – review and editing. **Tamirat Godebo Woyimo:** conceptualization, data curation, formal analysis, resources, validation, writing – review and editing.

## Ethics Statement

Ethical clearance was obtained from the Institutional Review Board (IRB) of College of Health Science and Medicine, Wolkite University.

## Consent

Written informed consent was obtained from the patient before starting the data collection process. The confidentiality and privacy of the patient were assured. Neither the data records nor the extracted data were used for any other purpose.

## Conflicts of Interest

The authors declare no conflicts of interest.

## Data Availability

The data that support the findings of this study are available from the corresponding author upon reasonable request.
